# Fluidic Response and Sensing Mechanism of Meissner’s Corpuscles to Low-Frequency Mechanical Stimulation

**DOI:** 10.3390/s25196151

**Published:** 2025-10-04

**Authors:** Si Chen, Tonghe Yuan, Zhiheng Yang, Weimin Ru, Ning Yang

**Affiliations:** 1 Fluid Machinery Engineering Technology Research Center, Jiangsu University, Zhenjiang 212013, China; spcyth9527@163.com (T.Y.); yzh13775745395@163.com (Z.Y.); ruweimin2005@163.com (W.R.); 2 School of Electrical and Information Engineering, Jiangsu University, Zhenjiang 212013, China

**Keywords:** Meissner’s corpuscle, low-frequency vibration, tactile receptor, flow field simulation, shear stress

## Abstract

Meissner’s corpuscles are essential mechanoreceptors that detect low-frequency vibrations. However, the internal fluid dynamic processes that convert directional mechanical stimuli into neural signals are not yet fully understood. This study aims to clarify the direction-specific sensing mechanism by analyzing internal fluid flow and shear stress distribution under different vibration modes. A biomimetic microfluidic platform was developed and coupled with a dynamic mesh computational fluid dynamics (CFD) model to simulate the response of the corpuscle to 20 Hz normal and tangential vibrations. The simulation results showed clear differences in fluid behavior. Normal vibration produced localized vortices and peak wall shear stress greater than 0.0054 Pa along the short axis. In contrast, tangential vibration generated stable laminar flow with a lower average shear stress of about 0.0012 Pa along the long axis. These results suggest that the internal structure of the Meissner corpuscle is important for converting mechanical inputs from different directions into specific fluid patterns. This study provides a physical foundation for understanding mechanotransduction and supports the design of biomimetic sensors with improved directional sensitivity for use in smart skin and soft robotic systems.

## 1. Introduction

Tactile perception is a fundamental component of the sensory system, responsible for transducing external mechanical stimuli into neural signals [1]. Among the diverse mechanoreceptors distributed throughout the skin, Meissner’s corpuscles assume a central role in sensing low-frequency vibrations within the 2–60 Hz range. Their dense distribution in glabrous skin, such as the fingertips, palms, and lips, appears to play a critical role in the high-resolution discrimination of fine textures and surface contours [2,3]. Structurally, Meissner’s corpuscles consist of nerve endings encapsulated by stacked lamellae of Schwann cells, a configuration that enables the near-instantaneous transduction of mechanical stimuli into bioelectrical signals, conferring properties of rapid adaptation and high sensitivity [4,5]. These characteristics establish them as a key biological archetype for engineering biomimetic prosthetics, virtual reality haptic interfaces, and systems for sensory nerve function assessment [6].

In recent years, advanced techniques have provided deeper insights into the function of Meissner’s corpuscles. Studies have employed selective genetic labeling to trace their development and functional dynamics (Neubarth et al., 2020 [7]), utilized in vivo dynamic imaging to visualize them in real-time (Pham et al., 2016 [8]), and used selective inhibition to confirm their critical role in fine tactile tasks, such as identifying micro-indentations or dehusking sunflower seeds (Zeng et al., 2018 [9]). More recently, principles from skin biomechanics have inspired the design of biomimetic sensors that mimic the corpuscle’s structure to investigate its stress-field sensing mechanisms (Shang et al., 2023 [10]). Collectively, these investigations highlight the intricate coupling between external mechanical stimuli, fluid dynamics, and the corpuscle’s microstructure. However, the differential mechanism by which distinct vibrational modes generate unique internal flow fields and shear stress distributions remains to be elucidated [11,12].

Most existing studies have focused on anatomical markers and behavioral assays, which can reveal the distribution and general function of Meissner’s corpuscles but are unable to provide direct measurements of fluid velocity or shear stress in the interstitial regions and along the receptor membrane surfaces [13]. These fluid dynamic properties are essential for membrane deformation and the activation of mechanosensitive ion channels [14]. The lack of this information restricts the understanding of direction-specific sensing mechanisms and limits the development of biomimetic systems based on Meissner corpuscle architecture [15,16].

To overcome this limitation, a biomimetic skin microfluidic platform was developed to replicate the structural features of the dermal papillae and Schwann cells in both geometry and spatial arrangement. This platform was integrated with a dynamic mesh method to simulate and experimentally analyze the flow fields under normal and tangential vibrations at 20 Hz. The results show that normal vibration leads to the formation of localized vortices along the short axis, while tangential vibration generates stable laminar flow and a planar shear field along the long axis. These observations support a direction-specific mechanism underlying low-frequency vibration sensing in Meissner corpuscles. The study provides a physical basis for understanding tactile signal transduction and supports the development of biomimetic sensors with directional sensitivity.

## 2. Materials and Methods

### 2.1. Design and Fabrication of the Microfluidic Platform

#### 2.1.1. Fabrication of the Microfluidic Chip

To simulate the microenvironment of Meissner’s corpuscles within the papillary dermis, a microfluidic chip was designed with a micropillar array that replicates the structural arrangement of dermal cells. This architecture recreates the local flow characteristics near the corpuscle and allows precise control of flow velocity and shear stress. The system provides an experimental platform for studying tactile response mechanisms under mechanical vibration.

The design of the chip was guided by the anatomical location and structure of Meissner’s corpuscles and the surrounding tissue. These corpuscles are located in the papillary dermis, which is less dense than the epidermis and reticular dermis. In this region, typical cell diameters are on the order of tens of microns [17]. To model this biological structure, micropillar diameters of 20 µm, 100 µm, and 200 µm were selected. These sizes represent the range of cell diameters and intercellular spacing near the corpuscles. The use of multiple scales reflects the diversity of cellular arrangements and enables evaluation of how the microenvironment influences shear stress and fluid flow. This is important for analyzing how vibrational stimuli propagate and are sensed across different structural scales. The use of three distinct pillar sizes also allows observation of flow behavior at various resolutions, improving experimental accuracy and control.

Additionally, to examine the influence of the lamellar structure of Schwann cells on tactile perception, the micropillar array was designed to replicate this arrangement. Two configurations were implemented: staggered and aligned. The staggered configuration closely resembles the natural stacked architecture of Schwann cells within Meissner’s corpuscles. This layout tends to produce asymmetric shear fields. In contrast, the aligned configuration represents a simplified pattern that forms straight transverse flow channels. This serves as a reference model for baseline fluid behavior and helps clarify how the more complex staggered structure influences flow velocity and shear stress. This design supports detailed analysis of the Schwann cell structure’s response to low-frequency vibration and forms a basis for studying the tactile sensing mechanism.

To recreate the authentic morphology of the papillary layer, the following assumptions were made: cells in the basal layer of the skin are spherical and closely packed. The spacing between adjacent cells is therefore defined as the distance between the centers of two spheres located on the space diagonal of a cube, given by:

(1) $$l=(\sqrt{3}-1)d$$

where ***l*** represents the intercellular spacing and *d* denotes the cell diameter (set to 20 µm), the calculated spacing is approximately 15 µm. However, due to limitations in photomask fabrication precision, the spacing in the micropillar array was controlled at approximately 20 µm. This value was selected to ensure structural fidelity while maintaining compatibility with fabrication constraints and flow-field visualization requirements. Although this 5 µm deviation differs from the ideal value, it is not expected to significantly influence the key fluid dynamic behaviors under study, such as vortex formation and direction-dependent shear stress. The chosen spacing preserves the essential geometric features of the biological structure and remains within an acceptable tolerance for capturing the core principles of mechanotransduction.

**Figure 1:**
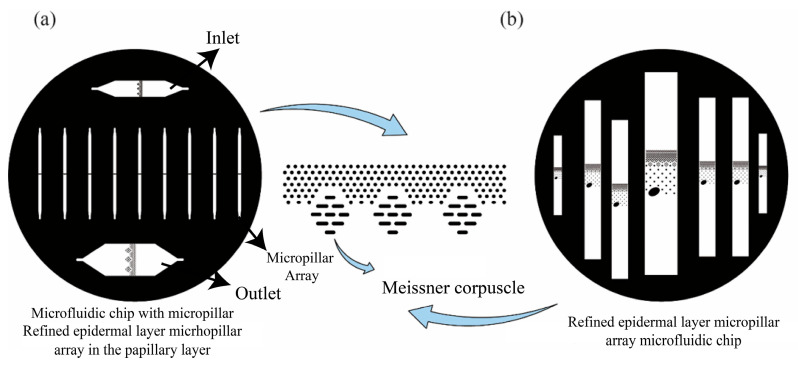
The design diagram of a microfluidic chip with micropillar arrays. (**a**) Papillary layer micropillar array structure; (**b**) Refined epidermal layer micropillar array structure.

To enable controlled morphological representation of the epidermal layers, the micropillar arrays were modeled according to the architecture of the stratum basale, stratum spinosum, stratum granulosum, and stratum corneum [18]. These layers were translated into arrays with varying densities and heights, forming a hierarchical microfluidic system that reflects the mechanical response pathway from the epidermis to the papillary dermis. As shown in Figure 1, the biomimetic chip includes two structural regions that recreate the skin microenvironment surrounding Meissner corpuscles. In Figure 1a, a staggered micropillar pattern mimics the lamellar arrangement of Schwann cells in the papillary dermis, which supports the generation of asymmetric flow fields. In Figure 1b, the epidermal layer is modeled by a vertically aligned pillar array with differences in height and spacing to represent the layered structure of the skin. These configurations were designed based on histological features to accurately reproduce interstitial fluid flow and shear stress distribution.

#### 2.1.2. Simulation of the Meissner Corpuscle Microenvironment

To simulate the microenvironment of the Meissner corpuscle, the micropillar array in the microfluidic chip was arranged in a staggered configuration. This layout replicates the lamellar structure of Schwann cells in the skin. The staggered design effectively recreates the irregular interstitial spaces between cells and allows fluid to flow through the interstitial matrix. This setup provides a platform to study how Schwann cell architecture influences local fluid dynamics, with a particular focus on its role in tactile perception under low-frequency vibration.

The working fluids used in the microfluidic chip were deionized water and fetal bovine serum (FBS) culture medium. Their respective dynamic viscosities were 1.003 mPa·s and 1.5 mPa·s, and their densities were 998.2 kg/m^3^ and 1020 kg/m^3^. Deionized water was selected to simulate the aqueous interstitial fluid of the skin, while FBS medium represented a more complex biological fluid environment. To enable flow visualization, both fluids were seeded with polystyrene fluorescent microspheres, which acted as tracer particles. The use of these fluids improves the biological relevance of the simulation and ensures that their dynamic behavior can be accurately modeled within the microfluidic platform [19].

For flow-field visualization, polystyrene fluorescent microspheres were used as tracers. The particle size was chosen to match the characteristic scale of the flow and the resolution of the microscopy system. Specifically, 200 nm diameter microspheres were used for chips with 20 µm micropillars, and 1 µm microspheres were used for the 100 µm and 200 µm micropillar arrays. The microsphere suspensions had a solid concentration of 2.5% (25 mg/mL) in all experiments. A syringe pump introduced the particle solution into the chip at a stable flow rate. Fluorescent particle movement was recorded using a CCD camera mounted on the microscope. The captured video frames were analyzed with Particle Image Velocimetry (PIV) software (PIVlab, v3.10, MATLAB R2023a) to calculate the flow field and produce velocity distribution maps. Details of the tracer particle selection are provided in Table 1.

### 2.2. Flow Velocity Measurement Using Micro-Particle Image Velocimetry (Micro-PIV)

**Figure 2:**
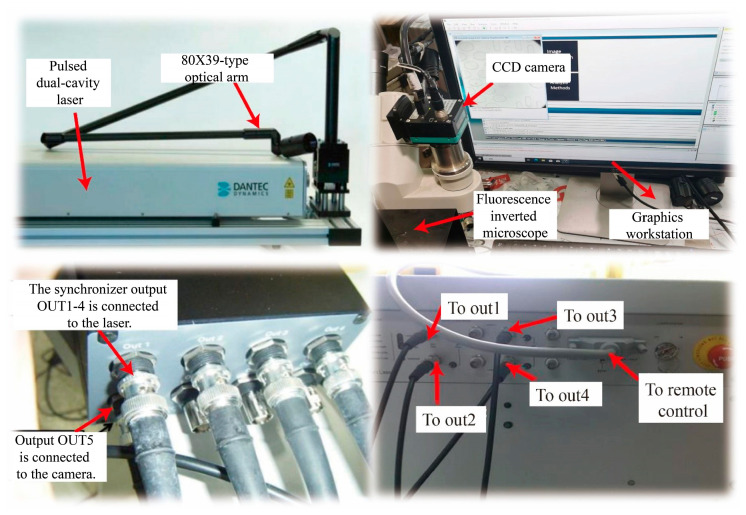
The Micro-PIV system and facilities.

The flow velocity field was quantitatively measured using a commercial Micro-Particle Image Velocimetry (Micro-PIV) system, as shown in Figure 2. The measurement setup consisted of a high-energy pulsed dual-cavity laser (38A7503, Dantec Dynamics, Skovlunde, Denmark), an inverted fluorescence microscope (Olympus IX73, Tokyo, Japan), and a high-resolution CCD camera (FlowSense EO 2M, Dantec Dynamics, Skovlunde, Denmark), all synchronized by a programmable timing unit (80N77, Dantec Dynamics, Skovlunde, Denmark). 

For the measurement, the tracer particles described in Section 2.1.1 were used at a solid concentration of 2.5% (25 mg/mL). The particle diameter was selected according to the scale of the micropillar arrays: 200 nm particles were used for chips with 20 µm pillars, and 1 µm particles were used for the 100 µm and 200 µm pillar arrays. The particle-laden fluid was introduced into the chip using a syringe pump at controlled inlet velocities ranging from 0.01 to 0.3 mm/min.

The measurement relied on a cross-correlation algorithm. Image pairs were captured and processed using Dynamic Studio v3.41 (Dantec Dynamics, Skovlunde, Denmark) to calculate particle displacement (Δx, Δy) over a known time interval (Δt), generating detailed velocity vector fields.

### 2.3. Dynamic Mesh Method and Vibration Simulation

To investigate the sensory mechanism of Meissner’s corpuscles under low-frequency vibration, a combined approach involving the dynamic mesh method and numerical simulation was employed. The dynamic mesh technique updates the computational grid in real time, allowing accurate simulation of Schwann cell displacement during vibration and the resulting fluid–solid interaction. This method is well suited for capturing the complex interplay between fluid motion and solid structures under low-frequency oscillatory conditions. It enables precise modeling of flow field changes induced by Schwann cell motion and their influence on tactile perception [20,21].

#### 2.3.1. Analysis of the Dynamic Mesh Method

The dynamic mesh method is primarily used to resolve flow-field variations caused by the vibration of the Meissner corpuscle. By simulating the oscillatory motion of Schwann cells, the method helps to clarify the influence of fluid dynamics on tactile perception. In the computational model, Schwann cells are treated as rigid bodies, and their surfaces are defined as moving boundaries. This framework describes the interaction between the fluid and the Schwann cells. Variations in shear forces at the fluid–solid interface directly affect the deformation of tactile receptors, which in turn influences sensory perception [22].

In this study, the dynamic mesh model is updated according to the following conservation equation [23]:

(2) $$\frac{\partial V}{\partial t}+(V\nabla )V=-\frac{1}{\rho }\nabla P+\frac{\mu }{\rho }\nabla^{2}V+F$$

where ***ρ***, ***µ***, and ***V*** represent the fluid density, viscosity, and velocity field, respectively; ***F*** is the external force field per unit volume; ***P*** is the pressure field per unit volume; and ***∇*** is the del operator.

Assuming the external force field is negligible, the equation can be further simplified to:

(3) $$\frac{\partial V}{\partial t}+(V\nabla )V=-\frac{1}{\rho }\nabla P+\frac{\mu }{\rho }\nabla^{2}V$$

The implementation of the dynamic mesh method within Ansys Fluent (v2020R1, ANSYS Inc., Canonsburg, PA, USA) is primarily based on three techniques: grid smoothing, local remeshing, and dynamic layering [24]. Grid smoothing is employed to adjust the mesh in the fluid domain, thereby maintaining mesh integrity and connectivity. Local remeshing, conversely, addresses regions where Schwann cell displacement is significant, which prevents mesh distortion and thus enables a more precise reproduction of the Schwann cell’s oscillatory motion.

Since the Schwann cell surface is defined as a moving boundary and experiences relatively large displacements during vibration, a combination of grid smoothing and local remeshing was applied to update the mesh. This strategy enables a more realistic simulation of the vibratory behavior of the Meissner corpuscle. The method accurately captures the evolving fluid–solid interactions and ensures that the effects of Schwann cell displacement on the flow field and shear forces are fully resolved [25,26].

A mesh independence study was performed to verify the accuracy and computational efficiency of the simulation. Six mesh configurations with increasing resolution were tested. The results showed that when the number of mesh nodes exceeded 1.6 million, the pressure drop between the inlet and outlet converged and remained stable at approximately 2.2 Pa. Based on this analysis, a mesh with around 1.65 million nodes was selected for all subsequent simulations to achieve a balance between accuracy and computational cost.

#### 2.3.2. Simulation Boundary Conditions and Motion Definition

Upon exposure to low-frequency vibration, tactile receptors within the Meissner corpuscle convert the mechanical stimulus into bioelectrical signals. These signals generate neural impulses that are transmitted to the brain, resulting in tactile perception. The lamellar structure of Schwann cells serves both to protect the encapsulated neurons and to maintain their spatial arrangement. To investigate the role of this structure in vibration sensing, a representative frequency of 20 Hz was selected from the low-frequency range (2–60 Hz), and the system’s responses to both normal and tangential vibrations were examined. Based on the relationship between frequency (*f*) and angular frequency (*ω = 2πf*), 20 Hz corresponds to an angular frequency of 130 rad/s.

The vibratory motion was defined using a User-Defined Function (UDF) written in the C programming language and compiled within the Ansys Fluent solver [27]. The UDF was implemented through the DEFINE_CG_MOTION macro to assign a sinusoidal velocity profile to the boundary walls of the Meissner corpuscle model. The displacement amplitude was set to 15 µm, ensuring that the velocity reaches 0 m/s at the maximum displacement. According to the dynamic mesh procedure described in Section 2.3.1, the grid smoothing option was enabled to maintain mesh quality during motion.

To verify the correct implementation of the prescribed motion, the displacement and velocity of the Meissner corpuscle were plotted as functions of time. The corpuscle first moved 15 µm in the positive direction, then accelerated through its original position to a displacement of 15 µm in the negative direction before decelerating to 0 m/s. This motion completed approximately five cycles within 0.25 s, corresponding to a vibration frequency of 20 Hz.

Compared to simulating vibration by adjusting the inlet flow rate, the dynamic mesh method more accurately preserves the structural constraints imposed by the cellular matrix and fibrous components on the interstitial fluid [28]. As a result, this approach more closely replicates the authentic flow field distribution surrounding a tactile receptor during mechanical stimulation.

## 3. Results

This study investigates the internal flow field characteristics of the Meissner corpuscle under normal and tangential low-frequency vibrations using a combination of numerical simulation and experimental observation. The dynamic mesh computational fluid dynamics (CFD) model successfully captured the interaction between the micropillar architecture, which mimics the lamellar structure of Schwann cells, and the surrounding fluid [29]. A vibration frequency of 20 Hz was selected to match the functional response range of the Meissner corpuscle. The system’s response to both vertical (normal) and horizontal (tangential) vibrations was examined, and the effect of different micropillar configurations on the resulting flow field was further analyzed. Flow behavior was visualized using velocity contours and streamline plots, while shear stress on the surfaces of the Schwann cell analogs was calculated to explore the mechanism by which vibration may trigger tactile activation.

**Figure 3:**
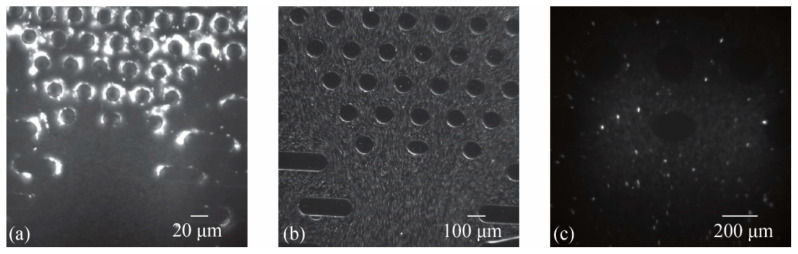
The distribution map of tracer particles in microfluidic chips. (**a**) 20 μm columns under 40× objective lens; (**b**) 100 μm columns under 10× objective lens; and (**c**) 200 μm columns under 10× objective lens.

Figure 3 shows representative snapshots of fluorescent tracer particle distribution within micropillar arrays of different sizes. These images present the raw visual data captured during the experiments. While each frame displays static particle positions, it is the sequential analysis of particle movement across frames that yields quantitative flow field information. This analysis forms the basis for generating velocity and streamline maps, enabling the investigation of fluid behavior around Schwann cells and its potential role in Meissner corpuscle function.

**Figure 4:**
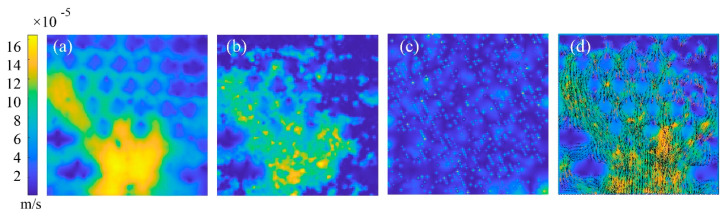
Velocity contour map during injection of a 10 mL syringe at a rate of 0.1 mm/min into a 100 μm micropillar chip. (**a**) Averaged velocity contour map; (**b**) Instantaneous velocity contour map; (**c**) velocity contour map with errors; and (**d**) Velocity vector map.

Building on the visualization shown in Figure 3, Figure 4 presents a quantitative analysis of the flow field derived from tracer particle motion. Using Particle Image Velocimetry (PIV), the particle distribution data were converted into a detailed velocity field. The figure illustrates multiple aspects of the fluid dynamics: the time-averaged velocity contour map (a) provides an overall representation of stable flow characteristics, while the instantaneous velocity field (b) and vector map (d) capture transient variations in flow magnitude and direction. This analysis is essential for validating the experimental methodology and transforming qualitative visual data into robust quantitative measurements. It forms the foundation for the subsequent evaluation of shear stress distribution and flow pattern evolution.

### 3.1. Validation of the Numerical Model

Before presenting the detailed simulation results, it is important to assess the reliability of the computational fluid dynamics (CFD) model. This was performed by comparing the simulated velocity profile with experimental data obtained from Micro-PIV measurements under identical geometric and flow conditions.

**Figure 5:**
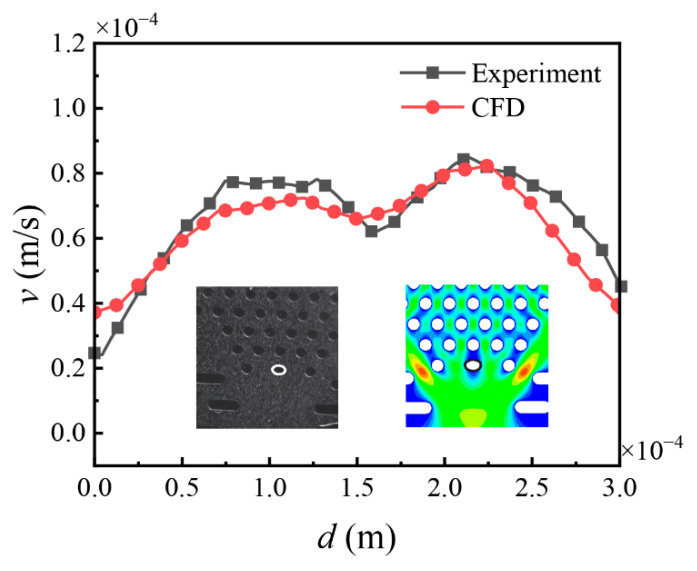
Numerical simulation and experimental verification.

As illustrated in Figure 5, the velocity profile was compared along a monitoring line positioned on the surface of an elliptical micropillar representing a Meissner corpuscle analog. The solid line represents the CFD simulation results, and the discrete markers indicate the corresponding Micro-PIV experimental data. A reasonable level of agreement was observed between the two datasets, with both the overall trends and velocity magnitudes appearing to be consistent. This level of consistency suggests that the numerical model is capable of capturing key features of the fluid dynamics in the microfluidic environment. Based on this comparison, the validated model was subsequently applied to investigate flow characteristics such as wall shear stress and vortex behavior, which remain challenging to access directly through experimental means.

### 3.2. Internal Flow Field Distribution During Normal Vibration

**Figure 6:**
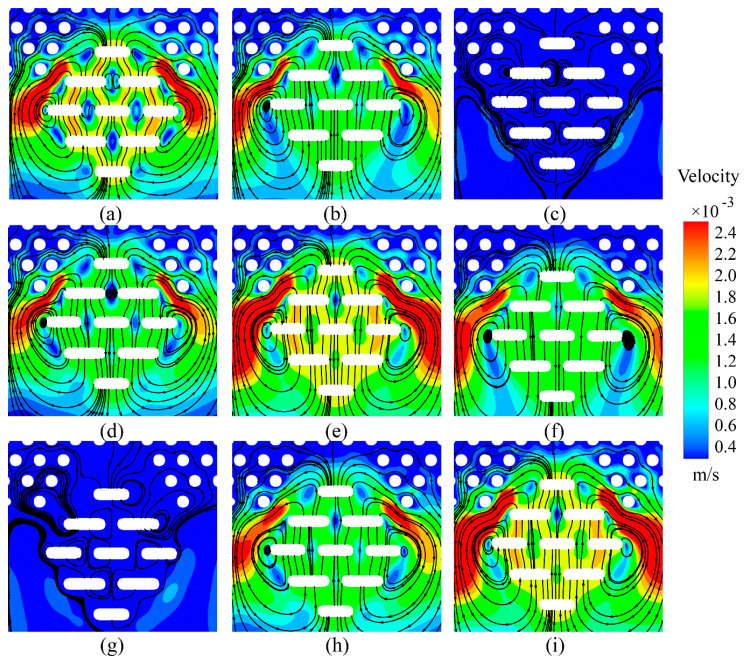
Velocity cloud and streamline maps inside the Meissner corpuscle during normal movement. The corresponding times are as follows: (**a**) 0T; (**b**) 1/8T; (**c**) 1/4T; (**d**) 3/8T; (**e**) 1/2T; (**f**) 5/8T; (**g**) 3/4T; (**h**) 7/8T; and (**i**) T; T is one period of vibration. The simulation reveals a peak fluid velocity of 2.38 × 10^−3^ m/s in the high-velocity zones during the cycle.

During normal vibration, the velocity of the interstitial fluid surrounding the Meissner corpuscle appears to be closely associated with the motion of the corpuscle itself. At peak displacement, where the corpuscle’s velocity momentarily reaches 0 m/s, the surrounding fluid becomes relatively quiescent, with only limited movement observed beneath the structure. In contrast, when the corpuscle moves at higher velocities, vortex-like structures tend to form along its lateral regions, as shown in the velocity contour and streamline plots in Figure 6. To illustrate the flow state more clearly, streamlines were superimposed on the velocity contours. Based on the streamline trajectories, panels (a), (b), (c), (h), and (i) represent the ascending phase of motion, while panels (d), (e), (f), and (g) correspond to the descending phase.

The array of Schwann cells within the Meissner corpuscle exhibits cohesive motion during vibration. Due to the corpuscle’s location directly beneath the epidermis, its displacement is partially restricted by the overlying tissue layer, leading to an asymmetric velocity distribution characterized by localized high-velocity regions in the superolateral areas.

Under normal vibration, where the micropillars representing Schwann cells are arranged in a staggered configuration, the streamlines are oriented primarily perpendicular to the micropillar layout. During the ascending phase of motion, this interaction may be approximated as a top-down compressive force acting on the skin. Under such conditions, a velocity gradient develops between the fluid regions above and lateral to the corpuscle, which appears to contribute to the generation of shear stress.

However, unlike the micropillar structure of the epidermis, the staggered arrangement of the Schwann cell analogs does not appear to form distinct high-velocity channels within the inter-pillar spaces. Instead, the flow velocity in these regions remains relatively low, and vortical structures are primarily observed at the ends of the micropillars. This behavior may be attributed to the spacing between the pillars and could help explain the differing responses of various tactile receptors to specific mechanical stimuli. These structural variations lead to distinct flow field distributions near the receptor membrane, which in turn may influence the perception of mechanical signals such as light touch and low-frequency vibration.

### 3.3. Internal Flow Field Distribution During Tangential Vibration

Under tangential vibration, the micropillar structure representing the Schwann cells in the Meissner corpuscle model is arranged in a parallel configuration, in contrast to the staggered layout used for normal vibration. This configuration results in a flow field distribution around the tactile receptor that appears noticeably different from that observed under normal vibration. The two models differ not only in the micropillar arrangement but also in certain geometric details. The inclusion of both configurations allows for a more comprehensive evaluation of the interstitial fluid’s response to directional vibrational stimuli.

**Figure 7:**
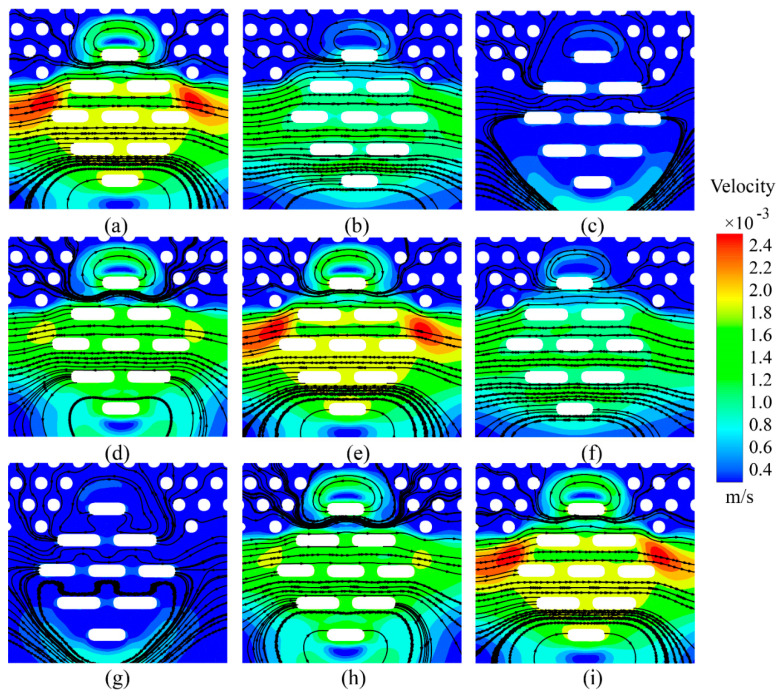
Velocity cloud and streamline maps inside the Meissner corpuscle during tangential movement. The corresponding times are as follows: (**a**) 0T; (**b**) 1/8T; (**c**) 1/4T; (**d**) 3/8T; (**e**) 1/2T; (**f**) 5/8T; (**g**) 3/4T; (**h**) 7/8T; and (**i**) T; T is one period of vibration. Under tangential stimulation, the flow exhibits a more laminar pattern with a peak fluid velocity of 2.15 × 10^−3^ m/s.

Figure 7 shows the velocity contours and streamline plots at various time points within a single vibrational cycle. Panels (a), (b), (c), (h), and (i) correspond to the rightward phase of motion, while panels (d), (e), (f), and (g) depict the leftward phase. Due to the influence of the surrounding papillary structure, localized high-velocity regions can be observed in the superolateral areas during vibration. A comparison of panels (a), (e), and (i) suggests that the flow velocity on the side corresponding to the direction of motion is slightly elevated, but the extent of the high-velocity region is smaller than that seen in the normal vibration case.

This difference appears to result from the more uniform arrangement of the micropillar array in the tangential vibration mode, which imposes less resistance to fluid flow compared to the obstruction introduced by the staggered configuration. As a result, the fluid–solid interaction is comparatively weaker. The streamline plots indicate that the fluid primarily moves along the interstitial gaps, forming an overall flow pattern that is more uniform and less disordered than that observed under normal vibration.

In the tangential vibration mode, the flow velocity within each horizontal layer remains generally stable, although slight increases in velocity can be observed within the interstitial regions. Notably, unlike in the normal vibration case, no near-static, low-velocity zones or clearly defined vortex structures were identified at the micropillar surfaces, which may suggest a more gradual and evenly distributed shear field.

### 3.4. Analysis of Wall Shear Stress on the Meissner Corpuscle

The visualizations presented in Figure 6 and Figure 7 illustrate qualitatively different flow patterns. To quantify these differences, peak fluid velocities were compared. Under normal vibration, the fluid is directed through more complex and tortuous paths, leading to the development of localized vortices and a peak velocity of approximately 2.38 × 10^−3^ m/s. In contrast, tangential vibration, which drives the structure along its long axis, results in a more ordered and laminar flow. This configuration imposes less resistance to fluid movement and is associated with a slightly lower peak velocity of approximately 2.15 × 10^−3^ m/s. These differences in both flow structure (vortices versus laminar) and velocity magnitude appear to contribute to the distinct shear stress distributions observed on the receptor surface, as discussed in the following section.

The above comparison suggests that the flow field within the microenvironment surrounding the Meissner corpuscle differs appreciably between the two vibration modes. In the case of normal vibration, the Schwann cell structure oscillates along its short axis, causing the fluid to follow irregular streamlines around the micropillars. This condition promotes vortex formation and localized turbulence. Conversely, tangential vibration along the long axis produces more uniform streamlines and results in a more focused momentum exchange across the interstitial region.

**Figure 8:**
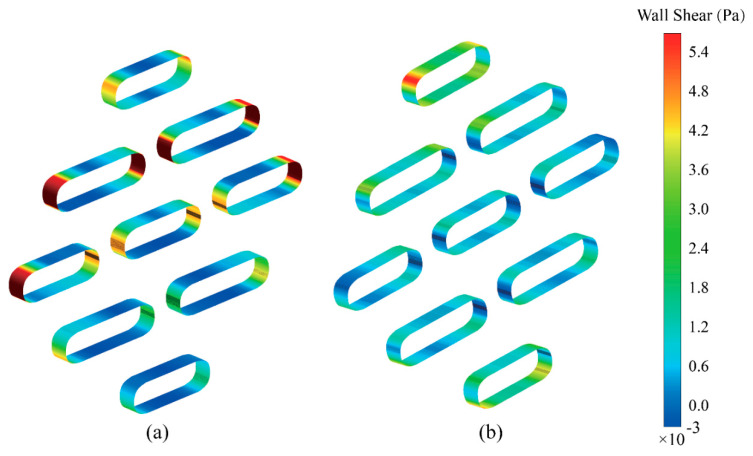
Wall shear stress distribution on the surfaces of the micropillars that represent the Schwann cell lamellae, captured at the moment of maximum fluid velocity. (**a**) Normal vibration, showing high shear stress concentrated on the short axis of the structure. (**b**) Tangential vibration, showing a more uniform but lower-magnitude shear stress along the long axis. The color scale indicates the magnitude of the wall shear stress in pascals (Pa).

To further quantify the mechanical effects of the two flow modes on the tactile receptor, the shear stress distribution on the surface of the Schwann cell analogs was calculated, as shown in Figure 8. The results indicate a clear quantitative difference. During normal vibration (Figure 8a), relatively strong hydrodynamic forces along the short axis produce elevated shear stress, with peak wall shear stress values exceeding 0.0054 Pa. In comparison, tangential vibration (Figure 8b), which is associated with smoother streamline patterns, yields a lower shear stress profile. The average shear stress along the long axis under tangential vibration is approximately 0.0012 Pa. This more than four-fold difference in peak shear stress magnitude between the two modes suggests the presence of a distinguishable mechanical cue that may support directional sensitivity in the tactile receptor.

Shear stress arises from velocity gradients between fluid layers, and its magnitude depends strongly on the local flow velocity distribution. Figure 8 illustrates the quantitative differences in wall shear stress under the two vibration modes. During normal vibration (Figure 8a), hydrodynamic forces along the short axis generate elevated shear stress, with peak values exceeding 0.0054 Pa on the surfaces of the micropillars. In contrast, tangential vibration (Figure 8b) is characterized by smoother streamlines and results in a lower average shear stress of approximately 0.0012 Pa along the long axis.

This more than four-fold difference in peak shear stress magnitude suggests the presence of distinct mechanical cues that may support the tactile receptor’s ability to differentiate between vibration directions. Specifically, this comparison provides insight into the directional sensing mechanism. It is possible that the sensory neurons within the corpuscle interpret the spatial distribution of shear stress to distinguish mechanical input. Higher, localized stress on the short axis may be associated with normal compressive stimulation, while a more sustained but lower-magnitude stress field along the long axis may encode tangential movement or slip. Such differential responses could contribute to the directional specificity observed in low-frequency vibration sensing.

Accordingly, the variation in flow patterns between vibration modes leads to spatially distinct shear stress distributions across the tactile receptor membrane, which may modulate its activation dynamics. These results suggest that the internal architecture of the Meissner corpuscle plays an important role in its sensitivity to subtle mechanical stimuli in the low-frequency range.

## 4. Discussion

This study proposes a potential physical mechanism underlying the sensory function of the Meissner corpuscle. The findings suggest that its internal structure may contribute to directional discrimination by generating two distinct fluid dynamic responses. Normal vibration induces localized vortices and elevated shear stress, whereas tangential vibration results in stable laminar flow with comparatively lower stress levels. These results extend beyond anatomical characterization and offer a fluid-mechanical framework for understanding tactile encoding.

The observed flow patterns and stress distributions may provide a physical basis for the known sensory behavior of the Meissner corpuscle. Elevated shear stress during normal vibration may be associated with the detection of initial contact and pressure by activating mechanosensitive ion channels. In contrast, the smoother and more sustained flow induced by tangential vibration may correspond to the detection of surface texture or slip. These observations suggest that the lamellar Schwann-cell sheath may function both as a protective layer and as a direction-selective mechanical filter for incoming stimuli.

This proposed mechanism has potential implications for the design of biomimetic tactile systems. Many existing tactile sensors are capable of measuring force magnitude but have limited ability to resolve its directional components [30]. The insights from this study suggest a design strategy in which microfluidic elements emulate the corpuscle’s internal architecture. Such an approach may enable electronic skin to passively distinguish between normal and shear forces. This capability could enhance robotic manipulation by detecting incipient slip and may improve tactile feedback in prosthetic and virtual reality applications [31].

This study was conducted at a representative frequency of 20 Hz. It is also relevant to consider how the observed fluid dynamic behaviors may vary across the broader functional range of the Meissner corpuscle (2–60 Hz). At lower frequencies (e.g., 5 Hz), reduced oscillatory velocity could allow the interstitial fluid to more closely follow the motion of the lamellae. This condition may produce gentler velocity gradients, resulting in weaker and less well-defined vortices, along with lower peak shear stress levels. In contrast, at higher frequencies (e.g., 50 Hz), the increased oscillation speed and associated fluid inertia are likely to create steeper velocity gradients, potentially giving rise to stronger vortices and elevated shear stress magnitudes.

This frequency-dependent variation in the fluid dynamic response may represent a potential mechanism for encoding frequency information. It is possible that the nervous system interprets shear-stress magnitude, together with the firing rate or signal intensity of activated sensory neurons, as indicators that contribute to the perception of vibration frequency [32]. A more pronounced and temporally concentrated mechanical signal may thus correspond to higher-frequency stimulation, offering a physical basis for frequency discrimination in tactile perception.

The current model is based on idealized assumptions, treating the cellular structures as rigid and the interstitial fluid as Newtonian. Future extensions of this work may benefit from incorporating the viscoelastic properties of biological tissue, which could influence the dynamic response of the system [33]. Specifically, the presence of viscoelasticity is expected to introduce several key effects. First, viscoelastic damping within the tissue may reduce peak shear stress levels, as part of the input energy would be temporarily stored and dissipated in the deforming matrix. Second, it may lead to more complex, phase-dependent behaviors. For instance, a temporal phase lag could emerge between the applied mechanical input and the resulting fluid flow, which may be relevant for encoding time-dependent features in tactile perception. Investigating these effects remains a valuable direction for future research [34]. A further step would be to integrate this fluid dynamic model with a neuronal activation framework to predict neural firing patterns. Ultimately, experimental translation into a physical sensor prototype would be necessary to evaluate performance and advance the development of tactile sensing technology.

Additionally, the present computational model represents a two-dimensional cross-section of the Meissner corpuscle. This 2D approach was selected to reduce computational cost while enabling detailed analysis of in-plane fluid dynamics. However, actual mechanotransduction processes occur in three dimensions. Out-of-plane flow effects, for example, may offer alternative fluid pathways, potentially decreasing the peak velocities and shear stresses observed in a confined 2D domain. Despite these limitations, the current model appears to capture the essential fluid–structure interactions underlying directional sensing. The core distinction between vortex-inducing compressive flow during normal vibration and the laminar flow associated with tangential motion arises primarily in the cross-sectional plane. Therefore, the 2D representation provides meaningful insights into how the corpuscle structure transduces directional mechanical stimuli into distinct fluid dynamic signatures [35].

In summary, this research connects cellular biomechanics with sensory neuroscience and proposes a fluid-based mechanism for directionally selective tactile encoding. These findings may contribute to the development of next-generation tactile sensors that mimic biological mechanoreceptors and enhance the performance of haptic systems in robotics, prosthetics, and wearable technologies.

## Figures and Tables

**Table 1:** Tracer sizes and inlet velocities used in the microfluidic experiments.

Microfluidic Chip Dimension (Micropillar Diameter)	Tracer Particle Diameter	Syringe Capacity	Inlet Velocity (mm/min)
20 μm	200 nm	1 mL	0.01, 0.12, 0.24
100 μm	1 μm	10 mL	0.1, 0.2, 0.3
200 μm	1 μm	10 mL	0.1, 0.2, 0.3

## Data Availability

The original contributions presented in this study are included in the article. Further inquiries can be directed to the corresponding authors.
